# Effects of Terlipressin on Management of Hypotensive Brain-Dead Patients Who are Potential Organ Donors: A Retrospective Study

**DOI:** 10.3389/fphar.2021.716759

**Published:** 2021-10-01

**Authors:** Donghua Zheng, Genglong Liu, Li Chen, Wenfeng Xie, Jiaqi Sun, Siqi Wang, Qiang Tai

**Affiliations:** ^1^ Intensive Care Unit, The East Division of the First Affiliated Hospital, Sun Yat-sen University, Guangzhou, China; ^2^ Department of Pathology, The Affiliated Cancer Hospital, Guangzhou Medical University, Guangzhou, China

**Keywords:** terlipressin, brain-dead, hypotension, organ transplantation, mean arterial pressure

## Abstract

**Background:** Administration of terlipressin can reverse hypotension in potential organ donors with norepinephrine-resistance. The aim of this study was to determine the effects of terlipressin on the hemodynamics, liver function, and renal function of hypotensive brain-dead patients who were potential organ donors.

**Methods:** A retrospective study was conducted by using the ICU database of one hospital. 18 patients in a total of 294 brain-dead cases were enrolled and administered terlipressin intravenously. All physiological parameters of recruited patients were obtained at baseline, 24 and 72 h after administration, and immediately before organ procurement.

**Results:** Terlipressin induced significant increases in mean arterial pressure (MAP) from 69.56 ± 10.68 mm Hg (baseline) to 101.82 ± 19.27 mm Hg (immediately before organ procurement) and systolic blood pressure (SBP) from 89.78 ± 8.53 mm Hg (baseline) to 133.42 ± 26.11 mm Hg (immediately before organ procurement) in all patients. The increases in MAP were accompanied by significant decreases in heart rate (HR) from 113.56 ± 28.43 bpm (baseline) to 83.89 ± 11.70 bpm (immediately before organ procurement), which resulted in the decrease of norepinephrine dose over time from 0.8 ± 0.2 μg/kg/min (baseline) to 0.09 ± 0.02 μg/kg/min (immediately before organ procurement). There were no changes in central venous pressure, liver function including aspartate aminotransferase (AST), alanine aminotransferase (ALT), and bilirubin. Renal function, assessed by serum creatinine (SCr), urine output (UOP), creatinine clearance rate (CCr), and estimated glomerular filtration rate (eGFR), improved significantly.

**Conclusion:** Our analysis of brain-dead patients with hypotension indicates that administration of terlipressin can significantly increases MAP, SBP, UOP, CCr, and eGFR, while decreases HR and Scr. Terlipressin appears to help maintain hemodynamic stability, reduce vasoactive support, and improve renal function.

## Introduction

Organ transplantation is considered as the optimal treatment for many patients with end-stage organ disease (Schnitzler et al., 2005; Weinrauch and D’Elia 2018). However, there is a widening disparity between the number of patients and the availability of suitable organs from brain-dead donors (Merchant et al., 2010; Matesanz et al., 2012). Thus, more than 10 critically ill patients die each day in Europe while waiting for organ transplants (Lesieur et al., 2014). Maintenance of the donor status after brain death may help to increase the number and quality of available organs (Linos et al., 2007). The challenge for physicians in the intensive care unit (ICU) is maintaining adequate organ perfusion and metabolism in brain-dead patients who are to become organ donors (Kotloff et al., 2015). Due to the great potential benefit from organ donation, optimal ICU management strategies should be applied to maintain viable organs after confirmation of clinical brain death.

There are many significant systemic changes during brain death, and hemodynamic instability is among the most notable. During the pathogenesis of brain death, after the hypertensive phase of the “catecholamine storm” (López et al., 2009), arterial tonus and heart inotropism eventually deteriorate, leading to hypotension and hypoperfusion. Unfortunately this hypotension is profound, sustained, and threatens organ viability (Dare et al., 2012). Continued hypotension leads to poor end-organ perfusion and inadequate tissue oxygenation. Thus, clinicians often administer vasoactive drugs including dopamine, norepinephrine and vasopressin to correct hypotension and maintain organ perfusion in organ donors.

Vasopressin is a nonapeptide hormone synthesized by the hypothalamus. Its active form arginine vasopressin (AVP) mediates vasoconstriction *via* V1 receptors, couples to phospholipase C, and increases intracellular Ca^2+^ concentration (Morelli et al., 2004). After brain death, a depletion of vasopressin often accompanies vasodilation shock, and administration of low dosage vasopressin can restore arterial pressure and reduce the necessary dosage of vasoactive drugs (Katz et al., 2000; Salim et al., 2005). Terlipressin is a synthetic analogue of vasopressin that has a similar pharmacodynamics profile, but different pharmacokinetic properties. In particular, the biological half-life of terlipressin is 6 h, while that of AVP is only 6 min (O’Brien et al., 2002). A small case study demonstrates that administration of terlipressin can reverse hypotension in potential organ donors with norepinephrine-resistance. The mean arterial pressure (MAP) increases in these patients, allowing reduction or withdrawal of norepinephrine (Piazza et al., 2012).

However, data on the efficacy of terlipressin infusion in brain-dead patients are limited, and the physiological function of liver and kidney is unknown. In this study, we evaluate the effects of terlipressin on hemodynamics, liver function, and renal function in potential hypotensive organ donors with brain death.

## Materials and Methods

### Patients

This study is a retrospective analysis by using the ICU database of the East Division of the First Affiliated Hospital, Sun Yat-sen University, which is a tertiary teaching hospital. We analyzed the records of brain-dead patients admitted between January 2015 and January 2018. The research protocol was compliant with the ethics committee norms of our institution, and due to its retrospective design, the committee abandoned the need for informed consent. The trial was registered with ClinicalTrials.gov (NCT03477461).

18 patients in a total of 294 brain-dead cases were enrolled, all of whom were potential organ donors—had been confirmed clinical brain death, but not yet by TCD, SLSEP, and EEG. In each case, the diagnosis of brain death was confirmed according to international standards (Wijdicks, 2001). Recruited brain-dead patients are hemodynamically unstable (MAP < 65 mm Hg), and require high-dose norepinephrine (>0.5 μg/kg/min) with fluid resuscitation to maintain MAP at 65∼105 mm Hg. The exclusion criteria were clinical diagnosis of obvious source of infection or sepsis, clinical diagnosis of diabetes insipidus (DI), or prior administration of vasopressin or a vasopressin analogue.

### Experimental Protocol

After confirmation of clinical brain death, when urine output <0.5 ml/kg/h following adequate fluid resuscitation and high-dose norepinephrine (>0.5 μg/kg/min), continuous infusion of low-dose terlipressin (0.02–0.06 μg/kg/min) were added according to the urine output. If there was no significant increase in urine output, the dose of the infusion of terlipressin should be increased (Dictus et al., 2009). After stabilization, the vasopressor agent (norepinephrine) was titrated down to maintain a MAP (65 mm Hg ≤ MAP ≤ 105 mm Hg) at a level determined by the attending intensive care physician. All donors were managed according to standardized guidelines, including the use of intravenous fluids and inotropic agents to maintain MAP (65–105 mm Hg), central venous pressure (CVP, 4–10 mm Hg), and urine output (UOP, 1–3 ml/kg/h); transfusion to maintain hemoglobin at 7.0 g/dl or more; electrolyte replacement; ventilator management to achieve partial oxygen pressure ≥90 mm Hg; and serum pH 7.35–7.45. Lung management included target tidal volume of at least 6 ml/kg ideal body weight; an inspiratory: expiratory ratio 1:1–1:2; early bronchoscopy to clear secretions; routine chest physiotherapy and pulmonary hygiene; prophylactic broad-spectrum antibiotic therapy; and manual recruitment maneuvers when indicated (Stoica et al., 2004).

Terlipressin was administered intravenously into all enrolled patients. All physiological parameters of recruited patients were obtained at baseline, 24 and 72 h after administration, and immediately before organ procurement.

### Parameters Investigated

Each patient had a systemic arterial catheter (Arrow International, Reading, PA) and a central venous catheter. Hemodynamic parameters, including heart rate (HR), mean arterial pressure (MAP), systolic blood pressure (SBP), and central venous pressure (CVP), were collected over time. The use of different vasopressor agents, such as norepinephrine and terlipressin, was recorded.

An indwelling urinary catheter was inserted into each patient, and urine was collected in a urinometer (Curity 0123, The Kendall Company, Hands, United Kingdom). The creatinine in 4 h urine samples (UCr) and in serum (SCr) were measured by an autoanalyzer using the Jaffe method. Measurements of UCr and SCr (mg/dl) and ultrafiltration (UF) rate (V, ml/min) were then used to calculate the creatinine clearance rate (CCr, ml/min) by using the formula: CCr = (UCr × V)/(SCr). The CCr was corrected for body surface area by dividing the calculated body surface area 1.73 m^2^. The estimated glomerular filtration rate (eGFR) was then calculated using the Modification of Diet in Renal Disease (MDRD) formula (Inker et al., 2018). The following laboratory parameters were also collected: sodium, osmotic pressure, lactate, brain natriuretic peptide, troponin I, aspartate aminotransferase (AST), alanine aminotransferase (ALT), bilirubin, SCr, and blood urea nitrogen (BUN).

### Statistical Analysis

All results are presented as means ± standard deviations (SDs). To evaluate the significance of changes over time in the systemic hemodynamic indexes, biochemical indexes, liver function indexes, and renal function indexes, an analysis of variance on repeated measures was performed for all quantitative variables, considering “time” as the “within” factor at the four measurements (baseline, 24 h, 72 h, and immediately before organ procurement).

All *p* values are two tailed, and a *p* value below 0.05 was considered statistically significant. Statistical analyses were performed using SPSS ver. 22.0 (SPSS Inc., Chicago, Ill., United States).

## Results

Table 1 summarized the clinical characteristics of the study group. 18 study objects were collected from total 294 brain-dead patients who were potential organ donors with hypotension, four of whom were female and 14 were male. Mean age were 38.1 ± 1.7 years, mean Acute Physiology and Chronic Health Evaluation [APACHE] II score were 28.6 ± 3.6 points, and mean Sequential Organ Failure Assessment [SOFA] score were 17.3 ± 2.6 points. 12 patients were brain-dead because of craniocerebral trauma, and the other six were because of intracranial bleeding.

**TABLE 1:** Donor baseline characteristicsby terlipressin exposure.

Variables	Terlipressin (*n* = 18)
Demographics	
Gender, male/female, n	14/4
Age, yr (mean ± sd)	38.1 ± 1.7
Cause of brain death	
Craniocerebral trauma, n	12
Intracranial bleeding, n	6
Co-morbidities, n	
Diabetes	0
Hypertension	1
Proteinuria	0
Disease severity (mean ± sd)	
Acute Physiology and Chronic Health Evaluation II	28.6 ± 3.6
Sequential Organ Failure Assessment	17.3 ± 2.6
Laboratory values(mean ± sd)	
Sodium, mmol/L	163.2 ± 16.4
Osmotic pressure, mOsm/L	349.7 ± 36.0
Lactate, mg/dl	2.7 ± 1.3
Brain natriuretic peptide, pg/ml	9122.9 ± 1512.5
Troponin I, mg/dl	0.5 ± 0.3
Aspartate aminotransferase, U/L	136.7 ± 78.0
Alanine aminotransferase, U/L	96.2 ± 58.9
Bilirubin, mg/L	25.4 ± 5.4
Serum creatinine, mg/dl	2.5 ± 1.2
Blood urea nitrogen, mmol/L	16.0 ± 7.2
Hemodynamic parameters (mean ± sd)	
Heart rate, beats/min	113.6 ± 28.4
Mean arterial pressure, mm Hg	69.6 ± 10.7
Systolic blood pressure, mm Hg	89.8 ± 8.5
Central venous pressure, mm Hg	13.2 ± 4.5
Urine production, ml/h	97.7 ± 38.7
Vasoactive agent (mean ± sd)	
Norepinephrine, μg/kg/min (*n* = 18)	0.8 ± 0.2

Table 2 showed the biochemical variables at different time points after terlipressin infusion. These data indicated significant improvements in sodium, osmotic pressure, and brain natriuretic peptide during the procedure. Sodium decreased from 163.2 ± 16.4 (baseline) to 147.0 ± 10.9 mmol/L (immediately before organ procurement) (*p* < 0.001), osmotic pressure decreased from 349.7 ± 36.0 to 325.6 ± 26.9 mOsm/L (*p* < 0.001), and brain natriuretic peptide decreased from 9122.9 ± 1512.5 to 5279.5 ± 1895.3 pg/ml (*p* < 0.001), while lactate and troponin I remained unchanged.

**TABLE 2:** Biochemical indexes at different points after terlipressin administration.

Variable	Measuring point				
	Baseline	24 h	72 h	Before organ procurement	*p* value
Sodium, mmol/L	163.2 ± 16.4^a^	155.4 ± 14.3^b^	147.9 ± 10.4^b^	147.0 ± 10.9^b^	<0.001^c^
Osmotic pressure, mOsm/L	349.7 ± 36.0^a^	338.6 ± 14.3^b^	330.8 ± 25.7^b^	325.6 ± 26.9^b^	0.001^c^
Lactate, mg/dl	2.7 ± 1.3	2.5 ± 1.2	2.5 ± 1.0	2.5 ± 0.9	0.701
Brain natriuretic peptide, pg/ml	9122.9 ± 1512.5^a^	7527.9 ± 2048.3^b^	5202.9 ± 3309.6^b^	5279.5 ± 1895.3^b^	0.001^c^
Troponin I, mg/dl	0.5 ± 0.3	0.4 ± 0.2	0.3 ± 0.1	0.2 ± 0.1	0.357

a Significant time effect.

b Significant effect versus baseline.

c *p* < 0.05 = significant.

Data are given as mean values ± SD.

**FIGURE 1:**
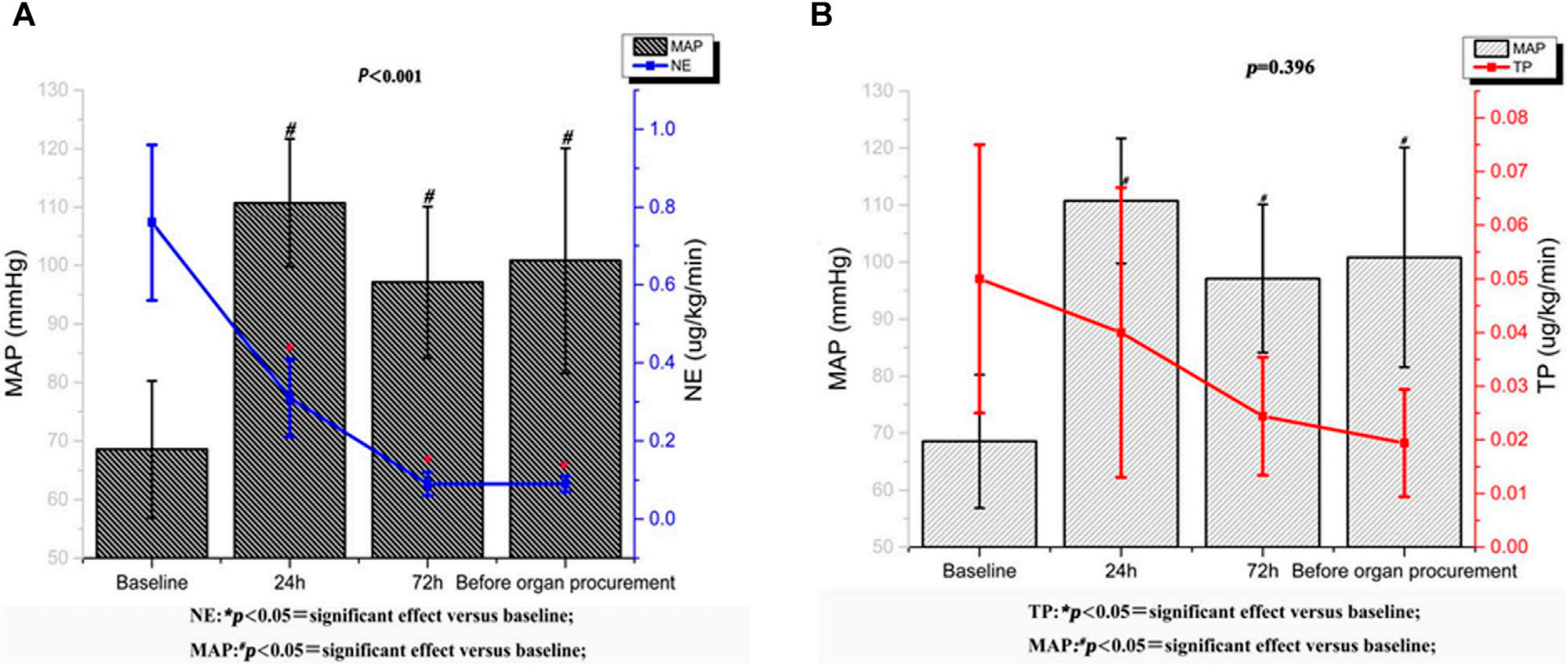
Norepinephrine and terlipressin requirement variations and mean arterial pressure trend after treatment with terlipressin.

Table 3 showed the values of the systemic hemodynamic parameters at different time points after terlipressin infusion. There were significant increases in MAP (69.56 ± 10.68 to 101.82 ± 19.27 mm Hg, *p* < 0.001), SBP (89.78 ± 8.53 to 133.42 ± 26.11 mm Hg, *p* < 0.001), and a significant decrease in HR (113.56 ± 28.43 to 83.89 ± 11.70, *p* < 0.001) in these brain-dead patients. The CVP declined slightly but not significantly. After administration of terlipressin, the dose of norepinephrine was decreased from 0.8 ± 0.2 μg/kg/min (baseline) to 0.09 ± 0.02 μg/kg/min (immediately before organ procurement), and the dose of terlipressin was decreased from 0.05 ± 0.01 μg/kg/min (baseline) to 0.02 ± 0.01 μg/kg/min (immediately before organ procurement) (Figures 1A,B).

**TABLE 3:** Systemic hemodynamic parameters at different points after terlipressin infusion.

Variable	Measuring point				
	Baseline	24 h	72 h	Before organ procurement	*p* value
Heart rate, beats/min	113.56 ± 28.43^a^	97.67 ± 20.14^b^	85.06 ± 13.51^b^	83.89 ± 11.70^b^	<0.001^c^
Mean arterial pressure, mm Hg	69.56 ± 10.68^a^	111.72 ± 10.98^b^	98.12 ± 13.00^b^	101.82 ± 19.27^b^	<0.001^c^
Systolic blood pressure, mm Hg	89.78 ± 8.53^a^	142.00 ± 14.17^b^	134.87 ± 17.77^b^	133.42 ± 26.11^b^	<0.001^c^
Central venous pressure, mm Hg	13.17 ± 4.35	14.39 ± 3.18	13.65 ± 3.43	13.53 ± 3.38	0.911
Norepinephrine, μg/kg/min	0.80 ± 0.20^a^	0.31 ± 0.10^b^	0.09 ± 0.03^b^	0.09 ± 0.02^b^	<0.001^c^

a Significant time effect.

b Significant effect versus baseline.

c *p* < 0.05 = significant.

Data are given as mean values ± S.

**FIGURE 2:**
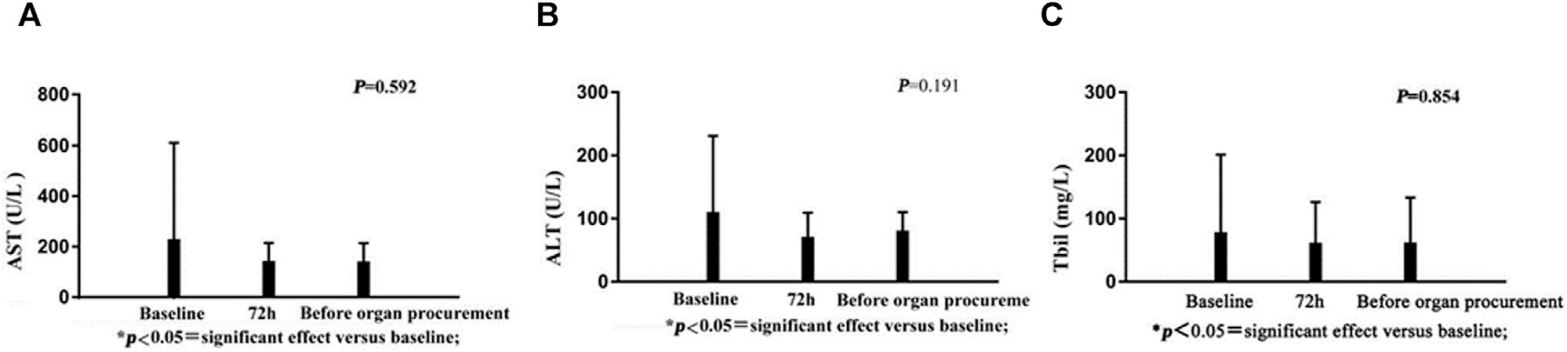
AST, ALT and Tbil during infusion of norepinephrine or terlipressin.

**FIGURE 3:**
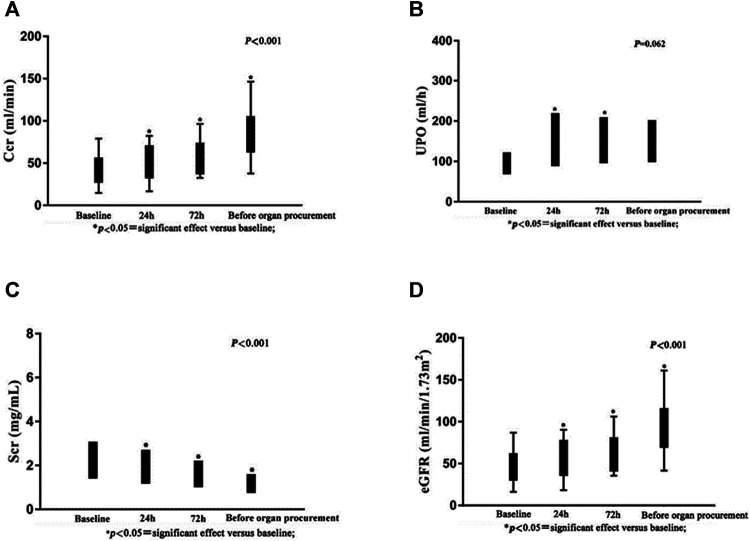
Serum creatinine, urine output, creatinine clearance rate and estimated glomerular filtration rate after intravenous bolus dose of terlipressin.

There were no significant changes in AST, ALT, or bilirubin following administration of terlipressin (Figures 2A–C). Urine output (UOP) increased above baseline levels after administration of terlipressin for 24 h, and was even higher at 72 h and maintained until organ procurement (Figure 3B). Terlipressin-induced increase of UOP was consistent with the significant increase of the creatinine clearance rate (CCr), eGFR, and the improvement of SCr (Figures 3A,C,D).

## Discussion

The major result of this retrospective study of hypotensive brain-dead patients who were potential organ donors is that terlipressin infusion significantly increases MAP, SBP, UOP, CCr, and eGFR, while decrease HR, SCr and the dose of norepinephrine. During the treatment, the dose of terlipressin could be decreased gradably from 0.05 ± 0.01 to 0.02 ± 0.01 μg/kg/min, the dose of norepinephrine was decreased from 0.8 ± 0.2 μg/kg/min (baseline) to 0.09 ± 0.02 μg/kg/min (immediately before organ procurement). Thus, terlipressin appears to improve hemodynamic stability, reduce vasoactive support, and improve renal function. As we know, it is the largest scale study of terlipressin on brain-dead donor.

The pathogenesis of brain death is characterized by failure of the autonomic nervous system and defects of several vasoconstrictor systems. Catecholamine deficiency and a transient “autonomic storm” lead to hypotension and diminished end organ perfusion (Chudoba et al., 2017; Squires et al., 2018). The goal of hemodynamic management in potential organ donors is to maintain optimal perfusion of all organs. Therefore, adequate volume expansion and vasopressor agents, including dopamine, norepinephyrine and AVP, are used to maintain the organ perfusion of brain-dead donors.

Chen et al. (1999) reports using low-dose continuous infusion of AVP (0.04–0.1 U/min) to increase MAP in patients who are hemodynamically unstable organ donors (MAP < 70 mm Hg), despite the use of catecholamine vasopressors. They find that low-dose AVP treatment significantly increase blood pressure and allow reduction or cessation of catecholamine administration. Currently, the Transplantation Committee of the American College of Cardiology recommends vasopressin as the preferred drug for stabilizing the hemodynamics in brain-dead heart donors (Souter et al., 2017). Unfortunately, vasopressin is unavailable in many countries, and terlipressin is licensed instead.

Terlipressin is a long-acting synthetic analog of vasopressin that has greater selectivity for V_1_ receptor than vasopressin which plays a vasoconstrictive role (Saad and Maybauer, 2017). As a prodrug, the body converts terlipressin into lysine vasopressin by cleavage of the N-triglycyl residue with endothelial peptidases, leading to a slow release of the vasoactive compound, lysine vasopressin (Choudhury et al., 2017). Terlipressin leads to contraction of the smooth muscles of internal vessels, reduces internal blood flow, and increases blood flow to major organs. It also reduces the concentration of plasma renin and increases renal blood flow perfusion, thereby increases GFR and improves renal function. Pharmacologically, terlipressin is superior to AVP in stabilizing hemodynamics and ensuring tissue perfusion (Rehberg et al., 2009). On the other hand, bolus injection of terlipressin may be associated with serious adverse reactions, including excessive micro-regional (such as lungs, coronary arteries, and internal organs) and systemic vasoconstriction, as well as decreased cardiac output and oxygen delivery. Compared with the “traditional” bolus injection, continuous infusion of low-dose terlipressin has beneficial hemodynamic effects and reduced side effects (Lange et al., 2011).

Our single-center, retrospective study of 18 brain-dead patients who are potential organ donors with hypotension indicates that terlipressin provides effective preservation of organ function in brain-dead patients who have unstable hemodynamics (MAP < 65 mm Hg). There are three major findings of our study. First, terlipressin infusion increases the MAP and SBP, and the increased MAP is accompanied by a significant decrease of HR. This effect remains until the organ procurement. Moreover, the dose of norepinephrine declines steadily and significantly over time. This is important because high-dose norepinephrine (>0.05 μg/kg/min) is associated with increased cardiac graft dysfunction. The extent and duration of catecholamine vasopressor therapy may contribute to the pathogenesis of adverse cardiac events, and should be avoided if possible (McKeown et al., 2012). Second, terlipressin administration markedly improves renal perfusion. According to Figure 3, after being treated with terlipressin for 72 h, CCR, SCR, and eGFR are improved significantly compared with no terlipressin or shorter (24 h) dosing time. It suggests that the optimal dosing time of terlipressin could be 72 h. Long-term use of vasopressors should be avoided in patients with hypotension and hypovolemia because, even though they improve blood pressure, they decrease renal blood flow and increase renal vascular resistance (Bellomo and Giantomasso, 2001). The effect of terlipressin is higher on efferent arteriolar resistance than on afferent arteriolar resistance, so it increases the filtration fraction and reestablishes urine flow (Narahara et al., 2009; Xiao et al., 2015). This may explain why administration of terlipressin to brain-dead patients increases glomerular perfusion pressure and flow, and hence GFR. Benck et al. report that administration of desmopressin (1-deamino-8-D-arginine-vasopressin, a synthetic arginine-vasopressin analogue) to brain-dead kidney donors is associated with improved graft survival (Benck et al., 2011). Because terlipressin and desmopressin have similar pharmacodynamic profiles, and terlipressin may improve the management of brain-dead patients who are potential organ donors, we expect that terlipressin probably also improves graft survival after kidney transplantation. Large and adequately powered prospective studies of this topic are warranted. Third, terlipressin does not significantly affect liver function, which is indicated by the levels of AST, ALT, and bilirubin. In contrast with terlipressin, AVP could cause an increase in bilirubin concentrations by reducing the biliary output and bile flow after an initial transient increase. These effects could be less pronounced when terlipressin is administered, probably due to its higher V1 selectivity (Hamada et al., 1992).

Several previous studies examine the efficacy of terlipressin as a pressor agent in solid organ donors with hypotension (Blasco et al., 2007; Vecchiarelli et al., 2010; Piazza et al., 2012). In particular, a study by Blasco et al. find that terlipressin restores MAP in brain-dead donors who had norepinephrine-resistant shock. This is consistent with our results. However, the effects of terlipressin on liver and renal function have not been investigated yet. Two other studies (case series) report the use of terlipressin in management of brain-dead patients but provide no statistical analysis (Hamada et al., 1992; Piazza et al., 2012).

There are several limitations of this study. First, because of the retrospective design, there is no suitable control group. Moreover, most of the potential donors are from traffic accidents, relative young age (mean age 38.1 ± 1.7 years), and only two causes of brain death (12 craniocerebral trauma and six intracranial bleeding), may not be representative enough. Thus, we cannot be certain that all the observed effects are due to terlipressin. It is possible that the conditions of all 18 patients would have improved spontaneously over time, although we believe this is unlikely. Second, we only examined 18 patients from a single institution, so the results are not necessarily applicable to other patient populations. Confirmation by a study with a much larger and diverse sample is necessary. Third, we focus on the effects of terlipressin in potential organ donors, and do not evaluate graft survival in recipients. Future studies should be extended to organ recipients, and include evaluation of graft function, incidence of primary nonfunctional or delayed recovery of graft function, and survival rates.

## Conclusion

The major results of this retrospective clinical study are terlipressin infusion could stabilize hemodynamic stability, reduce vasoactive support, and improve renal function in hypotensive brain-dead patients who are potential organ donors. In the future, a further properly powered, randomized, controlled trial is needed to confirm these results.

## Data Availability

The raw data supporting the conclusion of this article will be made available by the authors, without undue reservation.

## Abbreviations

ALT, alanine aminotransferase; AST, aspartate aminotransferase; AVP, arginine-vasopressin; Ccr, creatinine clearance rate; CrCl, creatinine clearance; CVP, Central venous pressure; DI, diabetes insipidus; eGFR, estimated glomerular filtration rate; HR, heart rate; ICU, intensive care unit; MAP, mean arterial pressure; MDRD, modification of diet in renal disease; SBP, Systolic blood pressure; Scr, creatinine; UOP, urine output.
